# Gene network modular-based classification of microarray samples

**DOI:** 10.1186/1471-2105-13-S10-S17

**Published:** 2012-06-25

**Authors:** Pingzhao Hu, Shelley B Bull, Hui Jiang

**Affiliations:** 1 Department of Computer Science and Engineering, York University, Toronto, M3J 1P3, Canada; 2 Prosserman Center for Health Research, Samuel Lunenfeld Research Institute of Mount Sinai Hospital, Toronto, M5G 1X5, Canada

## Abstract

**Background:**

Molecular predictor is a new tool for disease diagnosis, which uses gene expression to classify diagnostic category of a patient. The statistical challenge for constructing such a predictor is that there are thousands of genes to predict for the disease categories, but only a small number of samples are available.

**Results:**

We proposed a gene network modular-based linear discriminant analysis approach by integrating 'essential' correlation structure among genes into the predictor in order that the modules or cluster structures of genes, which are related to the diagnostic classes we look for, can have potential biological interpretation. We evaluated performance of the new method with other established classification methods using three real data sets.

**Conclusions:**

Our results show that the new approach has the advantage of computational simplicity and efficiency with relatively lower classification error rates than the compared methods in many cases. The modular-based linear discriminant analysis approach induced in the study has the potential to increase the power of discriminant analysis for which sample sizes are small and there are large number of genes in the microarray studies.

## Background

With the development of microarrays technology, more and more statistical methods have been developed and applied to the disease classification using microarray gene expression data. For example, Golub et al. developed a "weighted voting method" to classify two types of human acute leukemias [1]. Radmacher et al. constructed a 'compound covariate prediction' to predict the BRCA1 and BRCA2 mutation status of breast cancer [2]. The family of linear discriminant analysis (LDA) has been widely applied in such high-dimensional data [3-6]. LDA computes the optimal transformation, which minimizes the within-class distance and maximizes the between-class distance simultaneously, thus achieving maximum discrimination. Many other works have also extended the LDA framework for handling the large *p *(number of genes) and small n (sample size) problem. For example, Shen et al. developed an eigengene based linear discriminant model by using a modified rotated spectral decomposition approach to select 'hub' genes [5]. Pang et al. proposed an improved diagonal discriminant method through shrinkage and regularization of variance, a method to borrow information across genes to improve the estimation of gene-specific variance [6].

Studies have shown that given the same set of selected genes, different classification methods often perform quite similarly and simple methods like diagonal linear discriminant analysis (DLDA) and *k *nearest neighbor (kNN) normally work remarkably well [3]. However, because the data points in microarray data sets are often from a very high-dimensional space and in general the sample size does not exceed this dimension, which presents unique challenges to feature selection and predictive modeling. Thus, finding the most informative genes is a crucial task in building predictive models from microarray gene expression data to handle the large *p *(number of genes) and small n (sample size ) problem. To tackle this issue, different clustering-based classification approaches were proposed to reduce the data dimensions.

Li et al. developed cluster-Rasch models, in which a model-based clustering approach was first used to cluster genes and then the discretized gene expression values were input into a Rasch model to estimate a latent factor associated with disease classes for each gene cluster [7]. The estimated latent factors were finally used in a regression analysis for disease classification. They demonstrated that their results were comparable to those previously obtained, but the discretization of continuous gene expression levels usually results in a loss of information. Hastie et al. proposed a tree harvest procedure for find additive and interaction structure among gene clusters, in their relation to an outcome measure [8]. They found that the advantage of the method could not be demonstrated due to the lack of rich samples. Dettling et al. presented an algorithm to search for gene clusters in a supervised way. The average expression profile of each cluster was considered as a predictor for traditional supervised classification methods [9]. Similar idea was further explored by Park et al. [10]. They took a two-step procedure: 1) hierarchical clustering and 2) Lasso. In the first step, they defined super-genes by averaging the genes within the clusters; In the second step, they used the super-gene expression profiles to fit regression models. However, using simple averages will discard information about the relative prediction strength of different genes in the same gene cluster [9]. Yu also compared different approaches to form gene clusters and the resulting information was used for providing sets of genes as predictors in regression [11]. However, clustering approaches are often subjective, and usually neglect the detailed relationship among genes.

Recently, gene co-expression networks have become a more and more active research area [12-15]. A gene co-expression network is essentially a graph where nodes in the graph correspond to genes, and edges between genes represent their co-expression relationship. The gene neighbor relations (such as topology) in the networks are usually neglected in traditional cluster analysis [14]. One of the major applications of gene co-expression network has been centered in identifying functional modules in an unsupervised way [12,13], which may be hard to distinguish members of different sample classes. Recent studies have shown that prognostic signature that could be used to classify the gene expression profiles from individual patients can be identified from network modules in a supervised way [15].

In this study, we propose a network modular-based LDA (named as MLDA) method for improving the prediction performances of DLDA, DQDA and among others. The major difference between our method and other LDA-based methods is that MLDA incorporates the gene network modules into LDA in a supervised way. We built the MLD prediction model using modular-specific features. As a comparison, we also implement a variant of super-gene based regression models [10]. We first define super-genes by extracting the first principal component (PC) within the network modules. We then use the super-gene expression profiles to fit a logistic regression (LR) model. We named the method as MPCLR.

## Materials and methods

### Data sets

Three real microarray data sets are used in evaluating the performance of our proposed algorithm and other established classification methods. The detailed description of these data sets is shown in Table 1. We got the preprocessed colon cancer microarray expression data from http://genomics-pubs.princeton.edu/. For prostate cancer and lung cancer microarray data sets, we downloaded their raw data from gene expression omnibus (http://www.ncbi.nlm.nih.gov/geo/) and preprocessed using robust multi-array average (RMA) algorithm [16].

**Table 1:** Descriptive characteristics of data sets used for classification

Disease	Response type	No. Samples	No. genes/features	Reference
Colon cancer	Tumor/Normal	40 / 22	2000	[17]
Prostate cancer	Tumor/Normal	50 / 38	12635	[18]
Lung cancer	Tumor/Normal	60 / 69	22215	[19]

### Seed-based network-module identification

To identify gene modules in a gene co-expression network, we modify the correlation-sharing method developed by Tibshirani and Wasserman [20], which was originally proposed to detect differential gene expression. Specifically, we first use a seed-based approach to identify correlation-shared gene modules from gene network. Each of these modules includes a differentially expressed gene between sample classes, which is treated as a seed, and a set of other genes highly co-expressed with the seed gene. The revised approach works in the following steps:

**1**: Build a co-expression network using Pearson correlation coefficient (*r*) [21].

**2**: Compute test statistic *T_i_*(*i *= 1,2,..., *p*)for each gene *i *in the co-expression network using the standard t-statistics or a modified t-statistics, such as significance of microarrays (SAM) [22].

**3**: Rank the absolute test statistic values from the largest one to the smallest one and select the top *m *genes as seed genes.

**4**: Find the module membership *s *for each selected seed gene i* in the co-expression network. The module assignments can be characterized by a many to one mapping. That is, one seeks a particular encoder *C_r_*(i*) that maximizes

(1) $$i_{s}^{*}=\max_{\left\{0\leq r\leq 1\right\}}ave_{i\in C_{r}\left(i*\right)}|T_{i}|$$

Where $C_{r}\left(i*\right)=\left\{s:abs\left(corr\left(x_{i^{*}},x_{s}\right)\right)\geq r\right\}$. The set of genes *s *for each seed gene i* is an adaptively chosen module, which maximizes the average (*ave*) differential expression signal around gene i*. The set of identified genes *s *should have absolute (*abs*) correlation (*corr*) with i* larger than or equal to *r*.

### MLDA algorithm

We propose a new formulization of the traditional linear discriminant analysis. Specifically, we first use the seed-based approach to identify gene network modules. Then we perform LDA in each module. The linear predictors in all the identified modules are then summed up. The new modular-based classification approach returns signature components of tight co-expression with good predictive performance.

Let assume there are A and B two sample groups (such as disease and normal groups), which have *n_A _*and *n_B _*samples, respectively. The data for each sample *j *consists of a gene expression profile *x_j _*= (*x*_1*j*_,*x*_2*j*_,...,*x_pj_*), where *x_ij _*be the log ratio expression measurement for gene *i *= 1,2,...,*p *and sample *j *= 1,2,...,*n*, *n *= *n_A_*+*n_B_*. We assume that expression profiles *x *from group *k *(*k *∈ {*A*,*B*}) are distributed as *N*(*μ_k_*,∑*_k_*). The multivariate normal distribution has mean vector *μ_k _*and covariance matrix ∑*_k_*.

In a simplified way, we assume that ∑ = ∑*_A _*= ∑*_B _*= {*σ*_*i*,*i*'_}*i*,*i*' = 12,...,*p *, where$\sigma_{ii}=\sigma_{i}^{2}$,*σ*_*ii*' _= *σ_i'i _*and *σ_ii' _*is the pooled covariance estimate of gene *i *and gene *i' *for sample groups *A *and *B*. Therefore, when $\hat{\sum }$ is a block-diagonal structure, we have

$$\hat{\sum }={\left[\begin{array}{ccccc} {\hat{\sum }}_{1} & 0 & 0 & \ldots & 0\\ 0 & {\hat{\sum }}_{2} & 0 & \ldots & 0\\ 0 & 0 & {\hat{\sum }}_{3} & \ldots & 0\\ \vdots & \vdots & \vdots & \vdots & \vdots \\ 0 & 0 & 0 & \ldots & {\hat{\sum }}_{C} \end{array}\right]}_{p^{*}p}$$

where *C *is the number of blocks (gene modules) and${\hat{\sum }}_{c}$ is the estimated covariance matrix for block *c*(*c *= 1,2,...,*C*).

The linear predictor (LP) with block-diagonal covariance structure is given by

(2) $$LP=\overset{C}{\underset{c=1}{\sum }}{\left[x_{c}-\frac{1}{2}\left(\mu_{A}^{c}+\mu_{B}^{c}\right)\right]}^{T}{\hat{\sum }}_{c}^{-1}\left(\mu_{A}^{c}-\mu_{B}^{c}\right)$$

Where $x_{c}^{T}$ is the expression measurements of the genes in module *c *for a new sample to be predicted and $\mu_{k}^{c}(k\in \left\{A,B\right\}$ is the mean vector of the genes in module *c*. Obviously, linear discriminant analysis (LDA) and diagonal linear discriminant analysis (DLDA) [3] are the special cases of MLDA. That is, when *C *= 1, $LP={\left[x-\frac{1}{2}\left(\mu_{A}+\mu_{B}\right)\right]}^{T}{\hat{\sum }}^{-1}\left(\mu_{A}-\mu_{B}\right)$, where *x^T ^*is the expression measurements of *p *genes for a new sample to be predicted, so MLDA is simplified to LDA; when *C *= *p *(that is, each module has only one gene), $LP=\overset{p}{\underset{i=1}{\sum }}{\left[x_{i}-\frac{1}{2}\left({\hat{\mu }}_{A}^{i}+{\hat{\mu }}_{B}^{i}\right)\right]}^{T}\left\{\left({\hat{\mu }}_{A}^{i}-{\hat{\mu }}_{B}^{i}\right)/\sigma_{i}^{2}\right\}$, where *x_i _*is the expression measurement of gene *i *for a new sample to be predicted, so MLDA is simplified to DLDA.

We estimate the mean vector $\mu_{k}^{c}$ of the genes in module *c *as${\bar{x}}_{k}^{c}$ and use the pooled estimate of the common covariance matrix in each module *c*

(3) $${\hat{\sum }}_{C}=\frac{\left(n_{A}-1\right)S_{A}^{c}+\left(n_{B}-1\right)S_{B}^{c}}{n_{A}+n_{B}-2}$$

Where $S_{k}^{c}=\left\{{\hat{\sigma }}_{ii^{\prime }}^{c}\right\}$, i,i' = 1,2...,*p_c _*and *p_c _*is the number of genes in the module *c*. ${\hat{\sigma }}_{ii^{\prime }}^{c}$ is estimated as

(4) $${\hat{\sigma }}_{ii^{\prime }}^{c}=\left\{\begin{array}{cc} {\hat{\sigma }}_{i}^{2} & for\;i=i^{\prime }\\ {\hat{\sigma }}_{i}{\hat{\sigma }}_{i^{\prime }}{\hat{r}}_{c} & for\;i\neq i^{\prime } \end{array}\right)$$

Where ${\hat{r}}_{c}=median\left\{{\hat{r}}_{ii^{\prime }}\right\}$ i,i' = 1,2...,*p_c _*and i ≠ i', ${\hat{r}}_{ii^{\prime }}$ is the correlation estimate between gene *i *and gene *i*' in module *c *of sample group *k*.

∑_c _is inversible when *n *≥ *p_c_*, that is,

$$\overset{-1}{\sum }=\left[\begin{array}{ccccc} \sum_{1}^{-1} & 0 & 0 & \ldots & 0\\ 0 & \sum_{2}^{-1} & 0 & \ldots & 0\\ 0 & 0 & \sum_{c}^{-1} & \ldots & 0\\ \vdots & \vdots & \vdots & \vdots & \vdots \\ 0 & 0 & 0 & \ldots & \sum_{C}^{-1} \end{array}\right]$$

However, in some modules (say module *c*), it is possible that *n *<*p_c_*. In this case, ∑*_c _*is not inversible. We apply singular value decomposition (SVD) technology [23] to solve the problem. Assume ∑*_c _*is a *p_c _*×*p_c _*covariance matrix, which can be discomposed uniquely as ∑*_c _*= *UDV^T^*, where *U *and V are orthogonal, and $D=diag\left(\sigma_{1},\sigma_{2},...,{\sigma_{p}}_{{}_{c}}\right)$ with $\sigma_{1}\geq \sigma_{2}\geq ,...,\geq \sigma_{p_{c}}\geq 0$. If ∑*_c _*is a *p_c _*× *p_c _*nonsingular matrix (*iff σ_i _≠ *0 for all *i*(*i *= 1,2,...,*p_c_*)), then its inverse is given by $\sum_{c}^{-1}=VD^{-1}U^{T}$ where $D^{-1}=diag\left(1/\sigma_{1},1/\sigma_{2},...,1/\sigma_{p_{c}}\right)$.

The rule to assign a new sample *j *to group *k *is, thus, based on:$LP>=\log\left(\frac{n_{B}}{n_{A}}\right)$, sample *j *is assigned to group *A*; otherwise, it is assigned to group *B*.

### MPCLR algorithm

In order to compare MLDA with other super-gens based classification approaches, we also implement a variant of super-gene based regression models [10]. MPCLR classification algorithm includes three stages: 1) construct correlation-sharing based gene network modules; 2) extract meta-gene expression profiles from the constructed modules using principal component analysis (PCA); 3) classify samples using PCA-based logistic regression model. Here we briefly described each of the three stages:

*Stage 1: Construct seed-based gene network modules*. This can be done using the same approach as used in MLDA algorithm described above.

*Stage 2: Principal component analysis of correlation-shared expression profiles: *To do this, for each of the seed-based gene network modules, we perform principal component analysis. Specifically, for a given gene module with *p_c _*genes, we assume $x_{j}=\left(x_{1j},x_{2,j},...,x_{p_{c}j}\right)$ be expression indices of *p_c _*genes in the *j*th sample. Let ∑ be covariance matrix of *x *with dimension *p_c_xp_c_*. All positive eigenvalue of ∑ are denoted by $\lambda_{1}>\lambda_{2}>...>\lambda_{p_{c}}$ . The first PC score of the *j*th sample is given by $x_{j}^{*}=e_{1}^{t}x_{j}$, where *e*_1 _is the eigenvector associated with *λ*_1_. Therefore, we can define the super-gene expression profile for *n *samples in a seed-based gene module as $x^{*}\;=\left(x_{1}^{*},x_{2}^{*},...,x_{n}^{*}\right)$. The estimated values for the coefficient $e_{1}^{t}$(eigenvector) of the first PC can be computed using singular value decomposition (SVD) [23]. Briefly, assume *E *be an *nxp_c _*matrix with normalized gene expression values of *p_c _*genes in a given module, so we can express the SVD of *E *as E = *UDA^T^*, where *U *= {*u*_1_,*u*_2_,...,*u_d_*} is a *nxd *matrix (*d *= *rank*(*E*)), $D=diag\left\{d_{1}^{1/)2},d_{2}^{1/)2},...,d_{d}^{1/)2}\right\}$ is a *d × d *diagonal matrix where *d_k _*is *k*th eigenvalue of *E^t ^E*, *A *= {*e*_1_,*e*_2_,...,*e*_d_} is a *p_c_xd *matrix where *e_k _*is eigenvector of associated with *λ_k _*and coefficients for defining PC scores. Magnitude of loadings for the first principal component score can be viewed as an estimate of the amount of contribution from the module genes.

*Stage 3: Classification using PCA-based logistic regression model: *Assume *Y *is a categorical variable indicating the disease status (such as cancer or no cancer). Here we only focus on binary classification and suppose that *Y = 1 *denotes the presence and *Y = 0 *indicates the absence of the disease. Therefore, we can have the following supervised PCA-based logistic regression model:

(5) $$\log\left(\frac{p_{j}}{1-p_{j}}\right)=\beta_{0}+\overset{C}{\underset{i}{\sum }}\beta_{i}*PC1_{i^{*}j}+\varepsilon_{j}$$

Where $p_{j}=\mathrm{Pr}\left(Y_{j}=1|PC1_{i^{*}j},i^{*}=1,2,...,C\right)$. *PC*1_i__*j _is the first principal component score estimated from the seed gene module i* for sample *j *and represents the latent variable for the underlying biological process associated with this group of genes. The model was fitted using *GLM *function in stats R package.

### Comparisons of different supervised classification methods

We compared the prediction performances of MLDA with other established supervised classification methods, which include diagonal quadratic discriminant analysis (DQDA), DLDA, one nearest neighbor method (1NN), support vector machines (SVM) with linear kernel and recursive partitioning and regression trees (Trees). We used the implementation of these methods in different R packages http://cran.r-project.org/, which are *sma *for DQDA and DLDA, *class *for 1NN, *e1071 *for SVM and *rpart *for Trees. Default parameters in *e1071 *and *rpart *for SVM and Tree were used, respectively. For other methods (DQDA, DLDA, 1NN, MPCLR and MLDA), there are no tuning parameters to be selected. In the comparisons, seed genes were selected using t-test and SAM, respectively. We evaluated the performances of DQDA, DLDA, 1NN, SVM and Trees based on different number of the selected seed genes and those of MPCLR and MLDA based on different number of gene modules, which were built on the selected seed genes.

### Cross-validation

We performed 10-fold cross-validation to evaluate the performance of these classification methods. The basic principle is that we split all samples in a study into 10 subsets of (approximately) equal size, set aside one of the subsets from training and carried out seed gene selection, gene module construction and classifier fitting by the remaining 9 subsets. We then predicted the class label of the samples in the omitted subset based on the constructed classification rule. We repeated this process 10 times so that each sample is predicted exactly once. We determined the classification error rate as the proportion of the number of incorrectly predicted samples to the total number of samples in a given study. This 10-fold cross-validation procedure was repeated 10 times and the averaged error rate was reported.

## Results

Tables 2, 3 and 4 list the prediction performances of different classification methods applied to microarray gene expression data sets for colon, prostate and lung cancers, respectively. Here the different number of top seed genes (5, 10, 15, 20, 30, 40, 50) was selected by t-test. Since it is generally time-consuming to search for genes which are not only correlated with a given seed gene but maximize their averaged test statistic value (Formula 1), in order to save time, we only tested 10 cutoffs of correlation *r *from 0.5 to 0.95 with interval 0.05. We observed that the averaged correlation of genes in the identified modules is usually between 0.65 and 0.85 with the number of genes in the modules from 2 to 56, suggesting that the genes in the modules are highly co-expressed.

**Table 2:** Mean error rates of classification methods applied to colon cancer data set

No. genes	DQDA	DLDA	1NN	Tree	SVM	MPCLR	MLDA
5	0.113	0.113	0.210	0.226	0.113	**0.097**	**0.097**
10	0.177	0.177	0.161	0.290	0.129	**0.097**	0.129
15	**0.113**	0.129	0.129	0.242	0.145	**0.113**	**0.113**
20	0.145	0.129	0.161	0.258	0.129	**0.113**	0.129
30	0.145	0.129	0.161	0.194	0.145	0.129	**0.113**
40	0.145	**0.129**	0.145	0.210	0.145	**0.129**	**0.129**
50	0.145	0.145	0.194	0.226	0.145	0.145	**0.113**

**Table 3:** Mean error rates of classification methods applied to prostate cancer data set

No. genes	DQDA	DLDA	1NN	Tree	SVM	MPCLR	MLDA
5	0.227	0.239	0.261	0.227	0.216	0.216	**0.193**
10	0.205	0.193	0.284	0.318	**0.170**	0.193	0.182
15	0.250	**0.227**	0.261	0.295	0.261	0.239	**0.227**
20	0.216	0.227	0.250	0.273	**0.193**	0.205	0.205
30	**0.205**	0.216	0.239	0.295	0.216	0.216	**0.205**
40	0.261	0.250	0.295	0.318	0.250	0.261	**0.227**
50	0.227	0.227	0.341	0.330	0.216	0.250	**0.193**

**Table 4:** Mean error rates of classification methods applied to lung cancer data set

No. genes	DQDA	DLDA	1NN	Tree	SVM	MPCLR	MLDA
5	0.170	0.170	0.186	0.201	**0.162**	0.170	**0.162**
10	0.170	**0.147**	0.186	0.193	0.170	**0.147**	**0.147**
15	0.162	0.162	0.201	0.178	**0.132**	0.155	0.147
20	0.147	0.162	0.170	0.193	0.178	0.155	**0.132**
30	0.132	0.125	0.132	0.193	0.147	0.125	**0.116**
40	0.178	0.147	0.162	0.186	**0.132**	**0.132**	**0.132**
50	**0.125**	**0.125**	0.147	0.178	0.147	**0.125**	**0.125**

As we can see, the proposed MLDA has relatively better or comparable classification performances among all being compared classification methods in the three data sets. The performance of MPCLR is not consistent in the three data sets. This is likely that the variation in the given data captured by the first PC may be different. Other methods with better classification performances are DLDA and SVM. In general, all these methods except Tree works well for both colon and lung cancer data sets. The performances of these methods in prostate cancer data are slightly worse than those in colon and lung cancer data sets, which may be due to clinical heterogeneity among samples.

We also used SAM to select seed genes and evaluated their prediction performance using the same procedure as described above. Similar prediction results are observed as shown in Table 4. Overall, the MLDA has slightly lower error rate than other being compared classification methods (Table 5).

**Table 5:** Mean error rates of classification methods applied to lung cancer data set

No. genes	DQDA	DLDA	1NN	Tree	SVM	MPCLR	MLDA
5	0.178	0.170	0.193	0.225	**0.162**	0.178	0.170
10	0.170	0.170	0.209	0.193	0.178	0.155	**0.147**
15	0.186	0.147	0.201	0.225	0.146	0.132	**0.116**
20	0.147	0.162	0.186	0.178	0.186	0.155	**0.132**
30	0.147	0.178	0.132	0.193	**0.101**	**0.101**	**0.101**
40	0.178	**0.132**	0.178	0.186	**0.132**	**0.132**	**0.132**
50	0.162	**0.132**	0.162	0.186	**0.132**	0.155	0.147

In many cases, we found that the simple method DLDA works well. Its performance is comparable with the advanced methods, such as SVM. We also observed that the performances of predictors with more genes are not necessarily better than those of the predictors with fewer genes. For example, when t-test was used to select the seed genes, the best performance was obtained with only 5 genes for MPCLR and MLDA predictors in colon cancer data set (Table 2), 10 genes for SVM predictor in prostate cancer data set (Table 3) and 30 genes for MLDA predictor in lung cancer data set (Table 4). When SAM was used to select the seed genes, the best performance was also obtained with 30 genes for SVM, MPCLR and MLDA predictors in lung cancer data set (Table 5).

## Discussion and conclusions

In this study we developed a network modular-based approach for disease classification using microarray gene expression data. The core idea of the methods is to incorporate 'essential' correlation structure among genes into a supervised classification procedure, which has been neglected or inefficiently applied in many benchmark classifiers. Our method takes into account the fact that genes act in networks and the modules identified from the networks act as the features in constructing a classifier. The rationale is that we usually expect tightly co-expressed genes to have a meaningful biological explanation. For example, if gene A and gene B has high correlation, which sometimes hints that the two genes belong to the same pathway or functional module. The advantage of the method over other methods has been demonstrated by three real data sets. Our results show that the algorithm MLDA works well for small sample size classification. It performs relatively better than DLDA, 1NN, SVM and other classifiers in many situations. The modular LDA approach induced in the study have the potential to increase the power of discriminant analysis for which sample sizes are small and there are large number of genes in the microarray studies.

Our results are consistent with previous findings: The simple methods have comparable or better classification results than the more advanced or complicated methods [3]. This is likely due to the fact that there are more parameters to be estimated in the advanced methods than in the simple methods, while our data sets usually have much smaller number of samples than features/genes. We also tried to use more top genes (up to 100) in the classification models and similar result patterns (results were not shown) were observed as shown in Tables 2, 3, 4, 5. Although some previous studies showed that better results can be obtained when the number of top genes used in the prediction models are much larger than the number of samples, the improved performance may be due to over fitting effect. Moreover, for clinical purpose, it is better to include fewer number of genes rather than larger number of genes in the prediction models due to cost issues.

Previous studies have shown that the topological structure of a node (gene product) in a protein network is informative for functional module inference [21,24,25]. Moreover, some useful approaches have been developed to measure the topology similarity of pairs of nodes in weighted networks [21]. It will be interesting to explore the network topology-sharing based method rather than the correlation-sharing approach to identify seed-based gene network modules and place them into our network-based classification framework. The MLDA framework can be further extended in many ways. For example, it is possible to directly incorporate the modular-specific features in other advanced discriminant learning approaches (such as SVM). In the future we will explore these ideas in details.

## List of abbreviations

DLDA: diagonal linear discriminant analysis; DQDA: diagonal quadratic linear discriminant analysis; KNN: *k *nearest neighbor; LDA: linear discriminant analysis; LR: logistic regression; MLDA: modular-based linear discriminant analysis; MPCLR: Modular-principal component based logistic regression; PC: Principal component; RMA: robust multi-array average; SAM: significance of microarrays; SVD: singular value decomposition; SVM: support vector machines.

## Competing interests

The authors declare that they have no competing interests.

## Authors' contributions

PH designed and performed the analysis and wrote the manuscript. PH, SB and HJ designed the algorithms.
